# Engineering Microneedles for Therapy and Diagnosis: A Survey

**DOI:** 10.3390/mi11030271

**Published:** 2020-03-05

**Authors:** Liping Xie, Hedele Zeng, Jianjun Sun, Wei Qian

**Affiliations:** 1 College of Medicine and Biological Information Engineering, Northeastern University, Shenyang 110169, China; zhdl555555@gmail.com; 2 Border Biomedical Research Center, University of Texas at El Paso, El Paso, TX 79968, USA; 3 Department of Electrical and Computer Engineering, University of Texas, EI Paso, TX 79968, USA; wqian@bmie.neu.edu.cn

**Keywords:** microneedle, diagnosis, point of care, drug delivery

## Abstract

Microneedle (MN) technology is a rising star in the point-of-care (POC) field, which has gained increasing attention from scientists and clinics. MN-based POC devices show great potential for detecting various analytes of clinical interests and transdermal drug delivery in a minimally invasive manner owing to MNs’ micro-size sharp tips and ease of use. This review aims to go through the recent achievements in MN-based devices by investigating the selection of materials, fabrication techniques, classification, and application, respectively. We further highlight critical aspects of MN platforms for transdermal biofluids extraction, diagnosis, and drug delivery assisted disease therapy. Moreover, multifunctional MNs for stimulus-responsive drug delivery systems were discussed, which show incredible potential for accurate and efficient disease treatment in dynamic environments for a long period of time. In addition, we also discuss the remaining challenges and emerging trend of MN-based POC devices from the bench to the bedside.

## 1. Introduction

Wearable healthcare technologies cause significant consideration in publics and scientists, owing to the vast demand from medical laboratories and clinics [1,2]. According to the statistics, the market for wearable healthcare devices was $6.22 billion in 2017, which was expected to reach $14.41 billion by 2022, growing at a compound annual growth rate of 18.3% [3]. Wearable healthcare devices enable biological fluids to be treated and analyzed in the place near the patients, avoiding transporting the biological fluids to a specific laboratory or hospital [4]. Thus, wearable healthcare devices visibly narrow the time gap between sampling and diagnosis [5], eliminate the risk of sample contamination, and allow chronic patients to real-time monitor physiological functions at home [6]. Moreover, wearable healthcare devices contribute to the medical development in rural areas, where essential equipment and well-trained medical personnel are lacking [7].

Skin, as one of the most significant organs in human body, plays a crucial role in protection, perception, and secretion. Biofluids below the skin provide vital indications of human health [8]. However, stratum corneum as the outer layer of epidermis is a formidable barrier. Only small molecules (molecular mass <600 Da) can passively penetrate the skin [9]. Therefore, people usually utilize sharp devices to pierce the stratum corneum for sampling physiological fluids or delivering drugs [10]. However, transdermal sampling still causes pain and hollow hurt owing to the nature of traditional transdermal needles and syringes [11]. About 10% of the world’s population suffers from needle-phobia [12], which exposes them to a large health threat [13].

Fortunately, microneedles (MNs) with micro-scale sizes (generally ranging from 25 to 2000 μm in height) have been emerging as minimally invasive devices for transdermal sensing, sampling, and molecule delivery [9,14,15,16]. MNs with micro-scale sharp protrusions can pierce the stratum corneum, resulting in painless access to dermal layers. An appropriate length of MNs can avoid stimulating dermal nerve fibers or damaging dermal blood vessels. The tiny and shallow wounds resulting from the MNs can heal within 30 min [17]. These promising features reduce the horror of users and advance patient compliance. Therefore, MNs can serve as a new kind of point-of-care device, allowing for painlessly transdermal sampling, sensing, and drug delivery without the need for well-trained personnel [18]. Some long and fine MNs are applied to minimally invasive treatment of sensitive organs, such as the brain [19] and heart [20]. In addition, MNs devices are portable, which provides the possibility of continuous monitoring of human vital signs during daily life [21].

The preliminary study on MNs started in 1976, but it was not extensively exploited until the late 1990s because of advancements in microfabrication technology, which provided suitable tools for MNs manufacture. Up to now, MNs have been developed with various materials (e.g., silicon, glass, ceramic, metal, polymers, and carbohydrate) [22,23,24]. Besides, the customized structure design enables MNs to be suitable for specific applications. Over the last decade, MNs had been extensively applied to transdermal delivery of therapeutic compounds (e.g., insulin, proteins, DNA, vaccines, and cells) [20,25]. However, it is in recent years that an increasing number of studies have been reported to use MNs for transdermal diagnostic applications.

**Figure 1:**
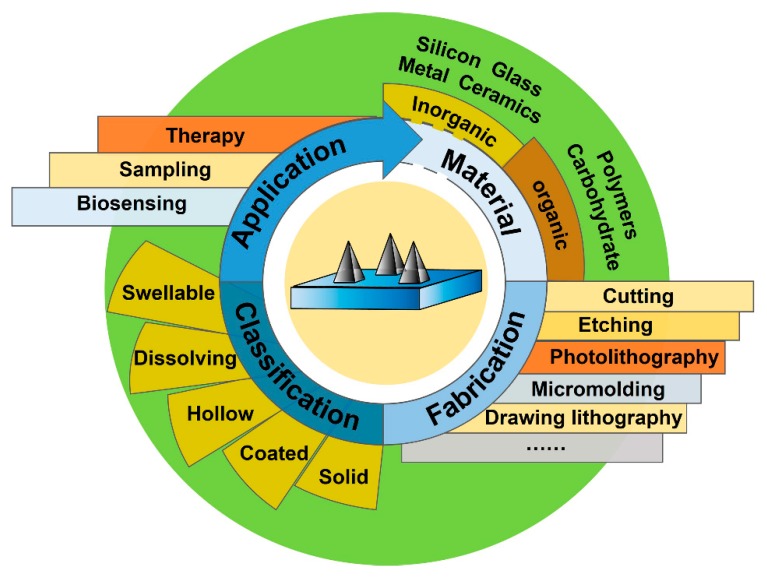
Schematic illustration of microneedles (MNs) commonly used in biomedical diagnosis and therapy (authors’ own work).

Many reviews on microneedle technology have been published in recent years. However, many of them typically focus on a specific aspect, such as materials science and manufacture [26], glucose monitoring [27], polymeric MNs’ application [14], diagnostics [28,29], and drug delivery [30,31]. The systematical review, which elaborates on the basic knowledge and main points of MNs, and covers the latest application of diagnosis and therapy, is still in its infancy [32]. This review aims to summarize the recent achievements in MN-based devices from materials, fabrication methodologies, and geometrical structures to applications (Figure 1). Commonly used critics for the design and performance of MNs are also discussed in context. In addition, the authors discuss the trend of trans-disciplinary integration of MN-based point-of-care (POC) devices for individual long-time monitoring and precise therapy. At last, challenge and outlook of MN-based POC platforms are discussed, indicating that the MN-based devices are promising tools in clinical application.

## 2. Materials and Fabrication Methods

### 2.1. Materials and Properties 

Materials decide the essential properties of MNs, such as toughness, flexibility, and permeability. Therefore, it is significant to choose appropriate materials for a special application. Various materials are used to fabricate MN devices. These materials can be classified into six categories: silicon, glass, ceramics, metals, polymers, and carbohydrate, as shown in Table 1.

#### 2.1.1. Silicon

Silicon is one of commonly used materials for MN fabrication owing to its excellent properties. Firstly, as the main material used in micro-electromechanical systems (MEMSs), silicon has excellent mechanical strength and biocompatibility [23,53]. These characteristics endow silicon-based MNs with better performance for diagnosis and therapy. Besides, because the MEMS fabrication technology is relatively mature [54], it is feasible to fabricate silicon-based MNs products with various lengths and shapes [33]. However, silicon costs a lot and needs expensive instruments to be processed into MN array [55]. In addition, the sharp wastes of silicon-based MNs may retain in the skin because of the brittle characteristic of silicon, which will induce topical inflammation [56].

#### 2.1.2. Glass

Glass has many remarkable properties including being transparent, cheap, and chemically inert. A relatively high Young’s modulus enables glass-based MNs to pierce the derma without deformation [57]. The chemical inertness of glass makes glass-based MNs safe in medical applications [38]. The low cost and simple fabrication method of glass-based MNs has drawn much consideration. However, glass is also brittle, which is dangerous. Additionally, its chemical inertness excludes it from almost all chemical fabricating methods, so glass-based MNs are still fabricated by the traditional micropipette pulling way [38]. The method can only handle one MN at a time, which cannot achieve a complex structure and needs manual operations.

#### 2.1.3. Ceramics

Ceramics, especially bio-ceramics, have been used in drug delivery for a long time. Porous ceramic-based MNs allow the drug to be incorporated in the interconnected pores and to diffuse directly after interposition [39,58,59]. Its inherent porosity endows the MNs with the ability to load drugs without extra fabrication process [60]. Moreover, by changing slurry components and sinter temperature, MN porosity can be adjusted to present different drug loading capacity and releasing speed [39]. Its biodegradable characteristic diminishes the risk of particle inflammation to a certain extent [61,62]. Meanwhile, a distinguished advantage of ceramic material is its stability in a high temperature and humidity environment [63]. However, ceramics-based MNs are significantly brittle. Tip fracture during insertion remains an unsolved problem. Besides, the material preparation and fabrication process of ceramics-based MNs usually needs more than 10 hours in total, which is troublesome and difficult for mass production [41,63].

#### 2.1.4. Metals

Metals are also used for the fabrication of MNs, because some metals (e.g., stainless steel and titanium) have outstanding mechanical strength and biocompatibility [42], while noble metals (e.g., silver, gold) usually work as sensitive components in a sensor [64]. Some nanostructured metal materials can present catalytic activity. However, because of its sharp tip and strength, the used metal-based MNs must be carefully treated before being discarded as biohazardous waste [65]. In addition, some metal materials can cause allergy [66], and the manufacture of metal-based MNs requires expensive MEMS fabrication techniques.

#### 2.1.5. Polymers 

Polymer is a versatile class of materials utilized in MN fabrication. According to their properties, polymer materials currently in use can be classified into two categories: degradable materials (e.g., gelatin, silk fibroin, polyvinylpyrrolidone (PVP), polyethylene glycol (PEG), polylactic acid (PLA), hyaluronic acid (HA), carboxymethyl cellulose, polyvinyl alcohol (PVA), poly(glycolic acid) (PGA), and poly(lactide-co-glycolide) (PLGA)) and non-degradable materials (e.g., polyacrylic acid (PAA), polydimethylsiloxane (PDMS), and polyvinyl methylvinyl ether (PMVE)). Polymer-based MNs can be fabricated in a variety of gentle ways, such as templating, microinjection, or low-energy graphic lithography at room temperature. Polymers-based MNs are particularly suitable for the delivery of active substances, such as vaccines, drugs, protein, and DNA. Polymers-based MNs with polymeric crosslinking network structure and hydrophilic groups (e.g., –NH_2_, –OH, –SO_3_H) possess swellability [67]. Some polymers are also highly regarded as substrate materials, because the relatively small Young’s modulus of polymer allows the substrate of MNs to be flexible, which allows MNs to adapt to some curved surfaces. On the other side, the low Young’s modulus of polymer also results in insertion failure. Therefore, a method should be considered to improve the mechanical strength of polymer-based MNs [68].

#### 2.1.6. Carbohydrate

Carbohydrates (e.g., maltose, chitosan, trehalose, sugar, dextran, raffinose, sucrose, mannitol, xylitol, galactose, and polysaccharide) can be employed to make MNs by micromolding [26,51]. Many carbohydrates are natural and have been used in medical care for a long time. Common fabrication approaches of carbohydrates-based MNs are micromolding and drawing lithography. Carbohydrate materials are usually used to fabricate dissolving MNs for drug delivery [38]. The carbohydrate-based MNs are able to pierce the stratum corneum owing to their relatively strong mechanical strength after solidification. In addition, carbohydrates are cheap and safe for human health. However, they have some drawbacks, such as mechanical failure after buckling, need for high processing temperatures, and extremely hygroscopic upon cooling [69,70].

### 2.2. Fabrication Methods of MNs

Various kinds of materials are applied to fabricate MNs. The materials mostly determine the methods for MN fabrication. Metallic MNs are usually fabricated by laser cutting, laser ablation, wet etching, metal electroplating, and micromolding [22,30]. Silicon MNs are commonly developed using wet etching, dry etching, and three-dimensional laser cutting [71,72]. Micromolding and sintering lithography are commonly employed in the preparation of ceramic MNs [73]. The fabrication method of polymer MNs involves micromolding, drawing lithography, photolithography, and droplet-born air blowing. Several commonly used methods are listed below.

#### 2.2.1. Cutting

Cutting is an efficient way to make uniform MN devices using a cutting machine. However, limited by material toughness and cutter size, the cutting accuracy is not high and the surface roughness of the fabricated MNs is not satisfactory [74]. In addition, the influence of blade wear on product quality should be taken into account when the blade is used for a long time [75]. The cutting method is usually suitable for materials with a certain degree of hardness, such as silicon and metal.

#### 2.2.2. Etching

As one of the basic fabrication methods for MNs, the etching method removes the specific part by the reaction between the etchant and substrate. The etching method can be divided into wet etching and dry etching according to the etchant. Wet etching uses liquid etchant to etch the substrate for obtaining the desired shape. Although limited by a few available materials and low selectivity, wet etching is still widely used in microfabrication [76]. It has relatively faster speed, and the etchants used in the wet etching are easier to treat compared with those in dry etching. Meanwhile, dry etching employs gas as the etchant, which has strong directivity and good etching effect, resulting in a relatively high resolution and potentially great etching depth. However, dry etching requires special equipment, high industrial cost, and special gas treatment to avoid ion bombardment. Regardless of wet and dry etching, most of the etching liquids (gases) are highly corrosive, and if the etching agent makes contact with the surface of the human body without protection, it will cause serious skin damage. Thus, etching operation requires strong professional skills. The etching methods are usually employed for the preparation of silicon MNs and metal MNs.

#### 2.2.3. Photolithography

**Figure 2:**
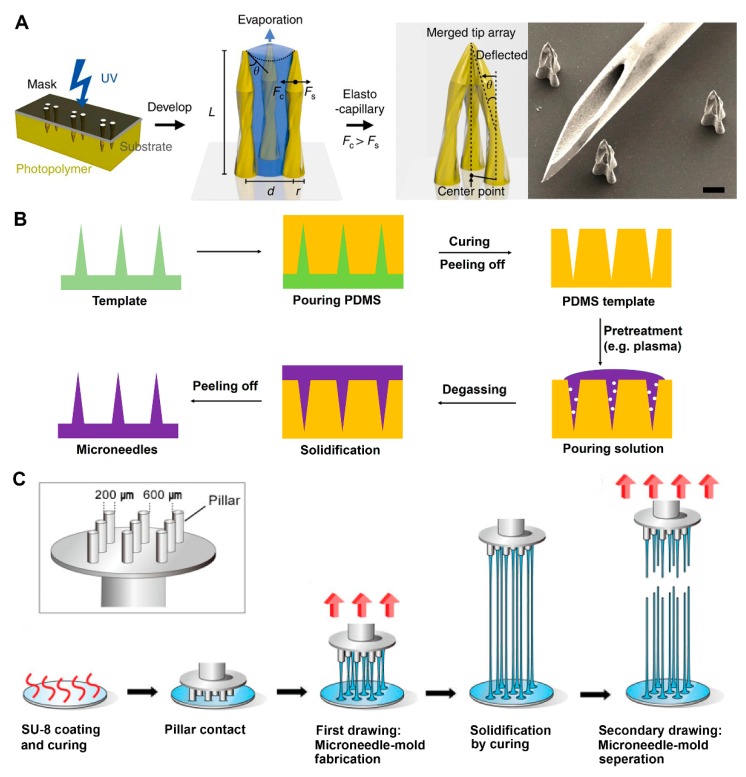
Schematic illustration of fabrication methods. (**A**) Procedure for the fabrication of a hollow MN by photolithography (reproduced with permission from the authors of [91]). (**B**) Typical process of MN fabrication via micromolding (authors’ own work). (**C**) Steps for producing primary MN array by drawing lithography(reproduced with permission from the authors of [88]). PDMS, polydimethylsiloxane.

Photolithography is a technique based on transferring a designed pattern from a photomask to a substrate with pre-coated photosensitive material using a radiation source, such as visible UV light or X-rays. With the aid of a set of well-defined control units, a 3D structure can be directly formed on the substrate [77]. The majority of photosensitive materials used in photolithography can be classified into two groups, positive resist and negative resist. The positive resist can be easily washed away by the developer after UV exposure, while negative resist will be reserved in the UV exposure place [26]. Typically, a refined photomask should be made before the whole procedure. The photomask is made up of flat transparent substrate and a patterned absorber layer. During the UV exposure period, the grayscale distribution of the absorber layer determines the illumination dose of different areas, subsequently resulting in 3D microstructure [78]. Except for application of the photomask, other alternative strategies, including switchable, movable laser source [77], and variable UV intensity [79], are also proposed to achieve 3D structure. Kathuria et al. proposed a single mold-free MN fabrication method. A Cr/Au coated glass slide was isotropically etched by HF/HCl to produce the microlens array. Then, a focused conical UV light path was achieved by the microlens array. The MNs assembled by the programmed light showed a sharp tip with a 14.7° angle in the short needle and 16.3° angle in the long needle, respectively [80]. Hollow MNs were developed by Lim et al. using photolithography (Figure 2A) [68]. A photomask with three transparent circles array and a polyethylene terephthalate (PET) film was put on the top of the photopolymer. After UV illumination, the photopolymer under these circles was polymerized, which became cured, and adhered to the medium PET film. The nascent pillars were immersed and washed in developer. Pillars appeared and merged in drying process owing to elastocapillary interaction. Although photolithography has been widely applied to fabricate MNs with desired performance, photolithography is usually applied to photopolymer. Young’s modulus of the photopolymer is relatively low, which highly limited its potential application to some extent.

#### 2.2.4. Micromolding

Micromolding employs a micro- or nano-scale mold to form MNs [81]. Typically, molten or liquid material is cast into the mold. Centrifugation or evacuation is required to remove air bubbles. MNs can be removed from the mold after solidification (Figure 2B) [82]. The micromolding technique provides a simple and quick solution for mass production [83]. The mild fabrication process is beneficial for bioactivity preservation of sensitive drugs, indicating that it is suitable for fabricating drug-loaded MNs [84]. However, conventional micromolding suffered from incomplete filling in the microstructure of the mold and remaining bubbles owing to the high viscosity of materials [85]. Normally, there are two strategies to overcome the problem. One is to improve the efficiency of the traditional degassing method, such as vacuum and centrifugation. For instance, Chen et al. developed a novel double-penetration female mold (DPFM) with the pinpoints covered by an air-permeable and hydrophobic membrane, thereby reducing gas resistance and conducting uniformly distributed MNs [86]. The base-to-tip gas exhaust and solution pouring were achieved synchronously, which was favorable in degassing. The method decreases the perfusion time from 20 min to 2 min, without a remarkable difference in insertion. The other strategy is adjusting the material or structure of MNs to decrease incomplete filling. McGrath et al. introduced an atomized spray technique into the micromolding procedure [87]. Single sugar composition was sprayed on the mold before perfusion as an amorphous layer to minimize the influence of liquid surface tension and viscosity. Significantly, MNs fabricated by this method had complete fidelity to its prototype, and presented feasibility on both sugars and polymers.

#### 2.2.5. Drawing Lithography

Drawing lithography was first proposed by Lee et al. in 2010 [88]. As shown in Figure 2C, instead of using conventional mold or masks, melted polymer can be drawn from 2D substrate to form a 3D needle-like structure directly. The cost of the whole procedure significantly declines without the need for UV illumination equipment. Additionally, drawing lithography overcomes the length and sharpness limitation of MNs, compared with other commonly used methods. These features make it possible to fabricate MNs on a large scale within a short time and provide a solution for long MNs fabrication [89]. However, drawing lithography also has demerits. Xiang et al. pointed out that drawing lithography was limited by alignment and material loading [50]. It was hard to produce dense, scale-up, and small MNs. In order to solve these problems, an improved self-loading drawing lithography method was proposed. An SU-8 membrane was attached to an SU-8 coated wafer to form a self-loading mold, which physically separates the SU-8 source and controls the source volume. The design successfully avoids the merging of the MN tips in the drawing process. The improved drawing lithography facilitated material reloading and prevented MNs from merging together, and thus achieved dense and scale-up MN fabrication. Besides, operations in drawing lithography are all based on flat substrates, which limited its application in complex structure fabrication. To address these issues, Chen et al. introduced magnetorheological fluid into drawing lithography. They added iron particles with a diameter of 1 μm to material as a magnet conductor, and an external magnetic field was applied to guide the self-shrinking procedure. The strategy significantly improved the sharpness and length of the MNs [90].

## 3. Classification of MNs

**Figure 3:**
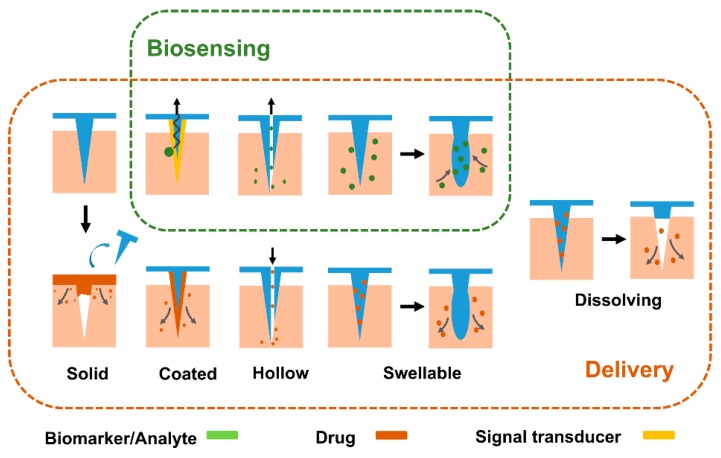
Schematic illustration of MN categories and their common application fields (authors’ own work).

Various types of MNs have been designed and utilized for transdermal sensing and therapy, including solid, hollow, coated, dissolving, and swellable MNs. Hollow, solid, coated, and swellable MNs patches are usually used for biosensing, while solid, coated, hollow, dissolving, and swellable MNs are mostly applied to drug delivery (Figure 3).

### 3.1. Solid MNs

Solid MNs are mostly applied to create microchannels in the skin. The pointed tips of MNs pierce the stratum corneum, and form micro size pores. Subsequently, the drug will enter the skin with less resistance if a drug patch is attached to the skin with microchannels, which enhances the passive absorption of drug [92]. In addition, solid MNs provide a simple way to extract interstitial fluid (ISF) by creating micropores and withdrawing dermal ISF assisted by an optional vacuum chamber. However, this method bears strong limitations for drug delivery and ISF extraction, which results in a high-risk pathway because it extends the time of occlusion and raises the possibility of microorganism infection [5,93]. While long sampling times (up to 20 min) are required to withdraw sufficient ISF. In addition, the skin perspiration will also be collected and will disturb analysis.

### 3.2. Coated MNs

Recent advancements of coated MNs enable a wide application, including bio-molecules delivery and sensing. Coated MNs can enhance dissolution of the bio-molecules (e.g., drug, protein, vaccines, and DNA) coated on the MNs [94]. The total amount of bio-molecules loading is dependent on the coating layer thickness and needle size. Coated layer containing enzymes or other catalysts allow MNs to act as a sensor for screening target quantitatively and selectively [95,96,97]. Biocompatibility and mechanical strength of coated MNs can be modified by a functional coating layer. A gold layer has been used for improvement of the biocompatibility and mechanical strength of MNs [98]. Biocompatible molecules such as PVP are applied to protect the probe layer [44].

### 3.3. Hollow MNs 

Hollow MNs with a vacant space inside are a variant of the conventional transfusion needle. Drug dispersion can be filled into the hollow MNs and deposited into the epidermis following MN insertion. An extra pump can be utilized to change the speed of drug solution carefully and refill the drug without removing the MNs, which makes continuous delivery of the drug possible [99]. Hollow MNs are also widely used to collect ISF [34] and blood [18]. In addition, a rapid analysis system can be precisely aligned on the outlet of the hollow MNs to discriminate analytes in the collected biological fluids (ISF or blood). According to this strategy, analytes (e.g., protein, ions, glucose in biological fluids) have been detection by hollow MN-based sensing systems. However, the chance of needle openings clogging during puncture of skin is a major limitation for hollow MNs, which will result in resistance to flow.

### 3.4. Dissolving MNs

Dissolving MNs are mainly used in drug delivery [100]. They are usually fabricated with biodegradable polymer or sugar, which endows the MNs with bio-acceptability and good patient compliance [101,102]. The tips of MNs can detach from the substrate of MNs upon contact with skin interstitial fluid. Drug substances encapsulated in the tips are released over time. The release time of drugs from the dissolving MNs ranges from minutes to days [103,104], which depends on the dissolution nature of polymers, fabrication process, geometry of MN, and water content of insertion part. Accordingly, release can possibly sustain for months by choosing appropriate types of polymer and fabrication methods [105]. Because of their biodegradability, dissolving MNs do not leave sharp biohazardous wastes in skin. It is much safer compared with solid and hollow MNs.

### 3.5. Swellable MNs

Swellable MNs are normally utilized in both drug delivery and biosensing owing to their excellent swellability [15,32,106]. When swellable MNs pierce skin, the biological fluids are absorbed into the MNs, owing to their polymeric crosslinking network structure and hydrophilic groups. Thus, the high concentration of drug in the MNs will be released to microcirculation [107,108]. In addition, MNs with swellability are able to collect ISF [109]. After a dry MN patch penetrates skin, ISF will be absorbed by the dry MNs through diffusion. However, an extra step (e.g., centrifugation) is needed to remove the analytes from the swellable MNs for further analysis [17]. Unfortunately, the lower mechanical strength of swellable MNs is the main weakness. It is wise to find a balance between the swellability and the mechanical strength by choosing appropriate polymers and fabrication processes.

## 4. Application of Microneedles

With the fast development of fabrication techniques, a wide range of MNs sizes and shapes can be fabricated using various materials. The materials and geometrical aspects directly affect the mechanical properties and performances of MNs. Clinically ideal MNs with good biocompatibility, strong mechanical strength, and cargos (such as proteins, peptides and vaccines) are highly desirable to achieve sensing or drug release in the skin. By rational design (including shape, aspect ratio, tip radius and length, and base width), MNs with excellent physical, chemical, and biological properties have been fabricated and utilized for applications of transdermal sensing, extraction of biofluids for diagnostics, and transdermal delivery [27,29,31].

### 4.1. Transdermal Sensing

The clinical relevance analytes (e.g., levels of glucose, biomarkers, and ion concentrations) are the key factors for disease diagnosis. Minimally invasive monitoring of these parameters both in peripheral blood and interstitial fluid (ISF) based on MNs is increasingly driven by the vast demand. ISF has many common components with blood and contents fluctuating in accordance with diseases. The concentrations of analytes in ISF can be used as indicators for the reflection of health status [110,111]. Furthermore, the less discomfort and lower skin inflammation rate of MNs compared with traditional needles contribute to high tolerability and acceptability of patients, which makes it possible to monitor health status continuously [112]. Some analytical techniques are commonly combined with MNs for quantitative detection of clinical relevance parameters.

#### 4.1.1. Electrochemistry 

**Figure 4:**
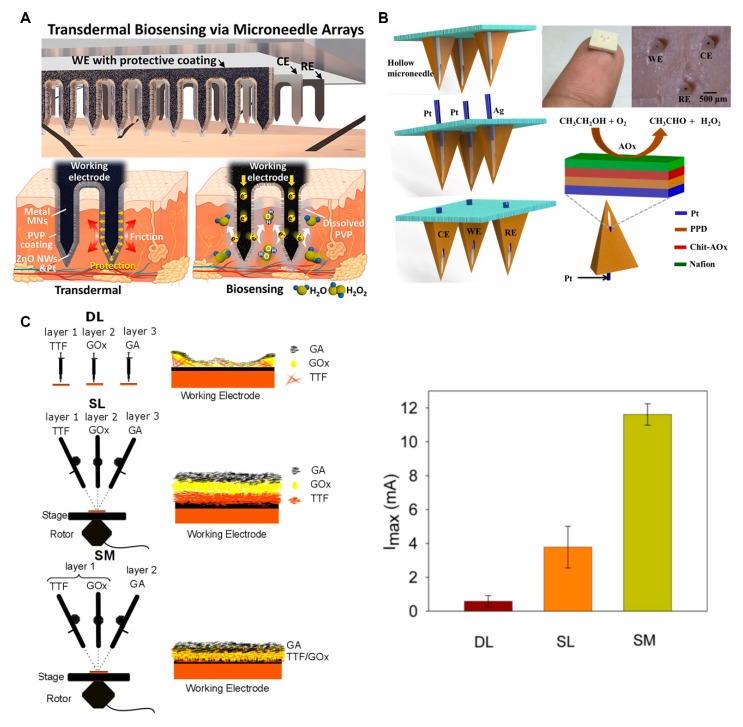
(**A**) Schematic illustration of dissolvable polyvinylpyrrolidone (PVP) layer/ZnO NWs/Pt nanostructure modified stainless MN. PVP coating provides protection during skin penetration, and rapidly dissolves after penetration, exposing sensitive probe for detection (reproduced with permission from the authors of [44]). (**B**) Schematic of a continuous minimally invasive alcohol sensor. (reproduced with permission from the authors of [21]) CE, counter electrode; WE, working electrode; RE, reference electrode. (**C**) Schematic presentation of three kinds of coating methods for MNs fabrication and average I_max_ of sensors gained by these methods in glucose monitoring experiment, which indicates the maximum limit of the amperometric current response and the maximum enzymatic reaction rate at the saturation point (reproduced with permission from the authors of [119]). DL, drop-cast method; SL, layer-by-layer method; SM, simultaneous spray mixing method; TTF, tetrathiafulvalene; GOx, glucose oxidase; GA, glutaraldehyde; PPD, poly(o-phenylenediamine); NWs, nanowires.

Electrochemistry is one of the popular ways employed in sensing. Chemical signals can be transformed into electrical signals for quantitative measurement. Oxidation-reduction as the key step of electrochemical measurements generates electron motion, which can transfer the concentration of target into electrical signal. Usually, there are two kinds of catalytic materials used in MNs, including enzymatic and non-enzymatic catalysts. Enzymatic catalysts usually possess high selectivity and catalytic effectivity, and are suitable for most biomedical applications. Representatives of non-enzymatic catalysts are nanomaterials including nanoporous Pt [113], Pt black [114], Pd nanoclusters [115], and so on. Compared with the enzymatic catalyst, the non-enzymatic catalyst has better stability, less environmental requirements, and is easier to store. Although there are some MNs with a naked sensitive layer [116,117,118], physical deformation (e.g., nanostructure collapse, enzyme detachment) during puncture into skin is still supposed to be eliminated. Liu et al. proposed a PVP barrier to protect MNs’ surface during penetration (Figure 4A) [44]. Raw stainless-steel MNs modified with ZnO nanowires (NWs) structure were used to improve surface specificity and enhance sensitivity. Pt layer with a thickness of ~20 nm was sputtered on the surface of the vertical ZnO NWs to transduce H_2_O_2_ concentration into electrical signal. PVP layer was coated on the surface to protect the inner nanostructure from damage during penetration. The PVP layer dissolved immediately once making contact with ISF, and the sensitive ZnO NWs/Pt structure was exposed to ISF for sensing. Owing to to the protection of the PVP coating, the device successfully preserved the integrity of nanostructures during skin insertion. As a result, the sensor showed three times higher sensitivity than the non-PVP-coated one, and could measure H_2_O_2_ in vitro ranging from 0 to 20 mM with sensitivity of 2.07 mA·cm^−2^·mM^−1^, which is excellent compared with other existing electrochemical sensors. Nafion membrane is widely used in MNs applications, which can avoid interference and provide long-term barrier protection owing to its ion selectivity. Chinnadayyala et al. demonstrated a Pt black-based MN sensor for continuous glucose monitoring [95]. Nafion and Pt black were sequentially deposed onto tips of Au-modified MNs, and were used as the working electrode for non-enzymatic sensing of glucose. The Au/Pt black/Nafion sensor showed linear range of 1–40 mM and detection limit of 23 ± 2 μM, which covered the physiological and pathological range of glucose. Furthermore, the result comparison between the MNs sensor and commercial glucometer in real serum sample test presented great compatibility. Mohan et al. further improved the membrane-based protection strategy and demonstrated an MN-based enzymatic electrochemical sensor for continuous alcohol monitoring (Figure 4B) [21]. A multilayer Nafion/poly(o-phenylenediamine) (PPD) coating served as an effective protective barrier, which kept potential interferents away from the sensor surface. Perm-selective Nafion membrane on the surface can protect the chitosan-alcohol oxidase (Chit-AOx) layer from negative charge contaminations. The size-exclusion PPD film under the Chit-AOx layer eliminated potential electroactive interferences. With these protections, the sensor showed attractive and stable analytical performance in continuous alcohol monitoring. 

Sensitivity is also a key factor for MN sensors. A general solution for improving sensitivity is to expand electroactive surface area and to accelerate the electron transfer rate. By modifying MNs surface with Au-multiwalled carbon nanotubes (MWCNTs) and methylene blue (MB), Bollella’s team successfully developed sensitive MNs sensors for the detection of lactate [112]. Au-MWCNTs were electrodeposited on the MNs’ surface as a base. Then, MB was deposited on the Au-MWCNTs film as mediator. Finally, lactate oxidase was immobilized on MB surface by the drop-casting method. Effective electron donor-acceptor interactions between Au-MWCNTs and MB endowed the sensor with outstanding electrocatalytic current and potential in the cyclic voltammetry test. The sensitivity of the sensor was nearly three times higher than the planar electrode, and had a large linear region from 10 μM to 200 μM with a 2.4 μM detection limit for detection of lactate. Research from Samavat’s group also showed the importance of mediator (Figure 4C) [119]. In their research, a water-insoluble tetrathiafulvalene (TTF) mediator was used to enhance the electron transfer from glucose oxidase (GOx) to the electrode. They applied different coating deposition techniques, such as the drop-cast method (DL), layer-by-layer method (SL), and simultaneous spray mixing method (SM), to functionalize MN electrodes for continuous glucose monitoring. Finally, glutaraldehyde (GA) was deposited on the top of the mediator to make sure GOx firmly cross-linked at this surface. The sensing layer gained by the SM technique possessed uniform TTF deposition and a large contact area between TTF and GOx, resulting in an order of magnitude improvement compared with the DL and SL method. The good mixing between TTF and GOx enhanced electrons transfer and achieved a good current response.

#### 4.1.2. Colorimetric Determination

**Figure 5:**
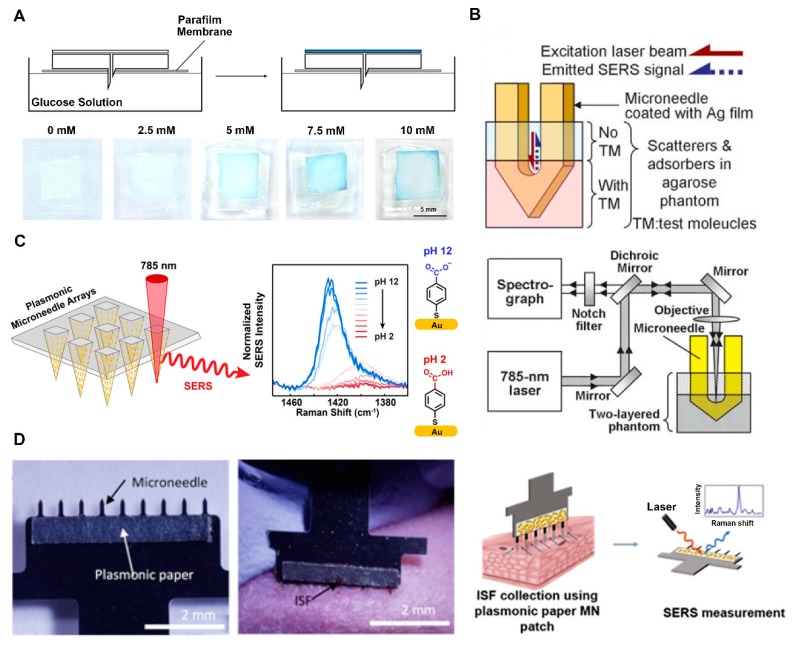
(**A**) Schematic illustration of a paper-based rapid colorimetric glucose sensor (top) and digital photographs of glucose-responsive backplates after addition of different concentrations of glucose(down) (reproduced with permission from the authors of [122]). (**B**) Schematic illustration of Ag-coated MN in two-layered human skin mimic (top) and Raman system for surface-enhanced Raman scattering (SERS) measurement (down) (reproduced with permission from the authors of [126]). TM, test molecules. (**C**) Schematic representation of plasmonic MN array for in situ pH measurement by SERS (reproduced with permission from the authors of [127]). (**D**) Photographs of MN patch with a plasmonic paper (left) and interstitial fluid (ISF) collection through MN penetrating into hairless rat (middle) and schematic illustration of SERS analysis of the plasmonic paper MN with target (right) (reproduced with permission from the authors of [128]).

Colorimetric analysis is a user-friendly method to visualize results and has been widely used in POC devices, owing to its low cost and no need for highly trained personnel. Normally, 3,3′,5,5′-tetramethylbenzidine (TMB) is popularly used in colorimetric tests as a chromogenic reagent. Peroxide released from enzymatic oxidation turns colorless TMB to blue color. The intensity of the blue color is related to the target concentration. Chen et al. developed a colorimetric method for early breast cancer detection [120]. Solid MN was adopted to insert into tumor tissue, while ISF was collected through a synthetic breathable thin membrane by siphon effect. An Ag_3_PO_4_/Ag-TMB system combined within the membrane will form a different depth of blue if carcinoembryonic antigen (CEA) presents in the ISF, thereby realizing colorimetric detection of breast tumors. Using this device, breast cancer can be diagnosed when cancer cells have not invaded into blood. The MN-based colorimetric system could detect the occurrence of breast cancers in seven days, which was only half of the classical blood test. This method remarkably advanced tumor detection time. In addition to an off-site test, MNs combined with a sensing component for on-site detection caters for monitoring chronic disease like diabetes. Mukerjee et al. utilized capillary tubes to extract ISF for blood glucose detection [121]. The ISF driven by capillary force could fill the microchannel automatically and be absorbed by the TMB-based detection strip. However, the saturation time of the colorimetric strip is about 15 minutes, which is too long for in situ diagnosis. Using the same reaction between TMB, enzyme, and glucose, Nicholas et al. used chromatography paper-based hollow MN to analyze glucose (Figure 5A) [122]. A square paper submerged in glucose-sensitive reagent in advance was attached to the output of the hollow MN as a backplate. This structure design obviously shorted the path length. The corresponding simulation experiment showed that the device can extract artifact ISF within five seconds, and the whole measurement can be finished in half a minute. Furthermore, diabetic patients can easily judge whether the blood glucose is higher than the normal blood sugar levels by discriminating the color intensity of the backplate, which makes it suitable for POC application.

#### 4.1.3. Surface-Enhanced Raman Scattering

Surface-enhanced Raman scattering (SERS) is a label-free non-invasive spectroscopic technique, arising from the combination of Raman spectroscopy’s fingerprint and localized surface plasmon resonances enhanced sensitivity [123]. Samples can be quantized by SERS without pre-treatment, which eliminates the sample dilution and centrifugation steps compared with conventional methods, resulting in a high processing speed and real-time measurement. Those features are appreciated for POC testing [124]. However, SERS is unsuitable to be applied directly to skin, because the laser penetration depth is around ~200 μm in Raman measurements [125]. Meanwhile, molecules of clinical interest mostly appear around the depth of 700 μm. MNs can pierce skin and its surface of the needles can be used as SERS substrate with simple modification. Yuen and Liu developed an Ag-coated MN probe based on intradermal SERS detection (Figure 5B) [126]. A 750 nm thick Ag film was coated on MN surface as the Raman scattering enhanced substrate. A two-layered human skin mimic containing Rhodamine 6G and glucose was made for evaluating the feasibility of the Ag-coated MN. A linear region from 5 to 150 mM was achieved by the device for glucose detection, indicating great potential for glucose monitoring. Park et al. reported a SERS-based transparent MNs patch for in situ pH detection (Figure 5C) [127]. 4-mercaptobenzoic acid (4-MBA) functionalized gold nanorods were immobilized on MNs surface as probes. 4-MBA is a pH-sensitive molecule that will be deprotonated at basic pH and protonated at acidic pH. Kolluru et al. combined a plasmonic paper with an MN patch for in vivo analysis of R6G from rats by SERS (Figure 5D) [128]. The plasmonic paper fabricated by immobilizing poly-(styrenesulfonate) coated gold nanorods on a thin strip of filter paper was used as SERS substrate. R6G in ISF collected by the MN patch was detected and quantified by calculating the intensity of SERS spectra from the plasmonic paper. Compared with conventional ISF analysis procedures, the SERS based method is quite simple, label-free, and low-cost.

### 4.2. Biofluids Extraction 

**Figure 6:**
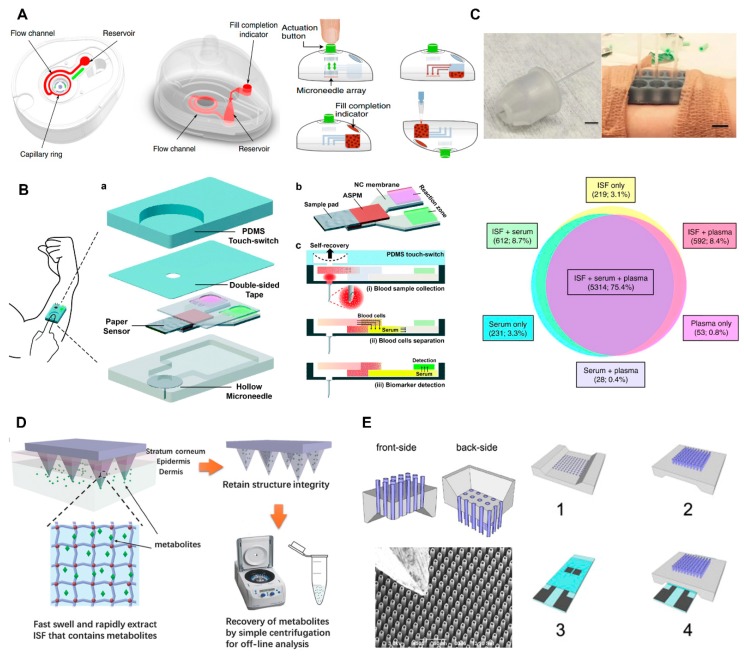
(**A**) Schematic illustration of a self-powered one-touch blood collector (left) and collection steps (right) (reproduced with permission from the authors of [34]). (**B**) Schematic illustration of one-touch-activated blood multi-diagnostic system. (reproduced with permission from the authors of [131]). ASPM, asymmetric polysulfone membrane; NC, nitrocellulose. (**C**) Hollow MN-based ISF collector (top-left) and MN holders attached to skin for ISF extraction (middle-left) and similarity of RNA species detected in ISF, serum, and plasma samples (down) (reproduced with permission from the authors of [133]). (**D**) Schematic of MeHA swellable MN designed for ISF collection (reproduced with permission from the authors of [17]). (**E**) Schematic illustration of silicon hollow MN-based ISF collector and its scanning electron microscope (SEM) image. Schematic illustrations including the front-side (1), back-side (2) of MN chip, real-time glucose measurement chip (3), and usage of the two chips (4) (right) (reproduced with permission from the authors of [34]).

Biofluids (e.g., blood, ISF) contain many useful substances that reveal much physiological and biochemical information. Screening of these biofluids is critical for the diagnosis of various diseases and evaluation of therapeutic efficacy. Blood collection devices, which are easy to use, safe, and fast for extracting essential tiny volumes of biofluids, play a significant role in POC diagnosis. However, conventional blood collection is painful, leads to bleeding, and requires well-trained professionals [129]. Microneedle provides an ideal transdermal biofluid extraction tool owing to its low cost, high safety, and painlessness. Hollow MNs and solid MNs are the main types of MNs used in blood extraction. Blicharz et al. invented a novel capillary blood collector (Figure 6A), which had received Food and Drug Administration (FDA) premarket clearance [18]. The device can yield a 100 μL blood sample painlessly through punctures in the skin created by solid MNs. The operation of the device is easy for common users by only pushing the single button of the device for blood collection. The extracted blood was mixed with anticoagulant automatically for diagnostic testing. Feedback from 144 participants supported that the device causes much less pain than that of conventional venipuncture. Hemoglobin A1c from device-collected capillary samples showed no significant difference from that from venous blood samples measured from the 144 participants, indicating minimal ISF contamination during the blood sampling process. However, the process of large volumes of blood being collected by MNs with fast speed is still in its infancy for POC application. Li et al. developed a self-powered one-touch blood collector [130]. The device was composed of a pre-vacuumed actuator and a hollow MN. A finger press induced negative pressure, driving blood samples to move along with the hollow MN. A total of 31.3 ± 2.0 μL of blood was successfully collected within 4 s from the ear artery of a rabbit with small resistance. The small blood volume is sufficient to meet the need of micro total analysis system (μTAS). Less than tens of microliters of the biofluids can be analyzed for further diagnosis [131]. The same group further integrated MN blood collector with paper-based microfluidic chip for in vivo glucose and cholesterol colorimetric measurement (Figure 6B) [131]. Blood collection, serum separation, and detection were all integrated into one device. The whole measurement including blood collection, serum separation, and screening analysis can be finished within 5 min, which is feasible to end-users as a POC device. It is worthy to notice that not all biomolecules or analytes from peripheral blood collected by MNs will be the same as that from the venous blood [132]. For some applications (e.g., glucose test, drugs of abuse, toxins, isolating DNA and RNA for sequencing and analysis), MNs are still accurate enough and can help the patient avoid venipuncture. For some tests, such as quantification of viral loads, it must be detected with venous blood.

Transdermal ISF including small molecules, electrolytes, and proteins, which strongly correlates with that of plasma, is an available proxy of blood for diagnosis [14]. Additionally, for cutaneous disease (e.g., melanoma, lupus), some specific biomarkers (e.g., exosome, resident memory T cells) are only or more enriched in ISF than blood [133]. Indeed, compared with blood, the sampling of ISF potentially causes less patient discomfort because of a drastic reduction of the insertion length, from 400–900 μm to 50–150 μm. Therefore, MN is a promising tool for transdermal ISF collection. A sharp tip and tiny size allow MNs to accomplish this mission without the hurt of pain-sensing neurons. Various types of MNs including hollow MNs, solid MNs, and swellable MNs have been reported to be used in ISF extraction. Hollow MNs rely on capillary action for collection of ISF, while swellable MNs depend on fluid diffusion and material absorption [28]. Because of its polymeric crosslinking network structure and hydrophilic groups, it can swell and absorb ISF. Miller et al. collected ISF and analyzed exosome, protein, and RNA from the collected ISF using hollow MNs (Figure 6C) [133]. ISF was proved to profile dynamic RNA-seq change, determine transcriptome, and proteome signatures. Chang et al. can collect 1.4 mg ISF in 1 min for metabolic analysis using novel MeHA swellable MN (Figure 6D) [17]. ISF absorbed by swellable MNs was recovered by centrifugation for off-site measurement. Glucose and cholesterol detected by this method showed a similar trend compared with the gold standard. ISF can also be analyzed in situ by integrating a collector with a sensing element. Takeuchi et al. reported a swellable MN array attached to a μTAS device for screening of glucose from ISF [134]. Strambini et al. measured glucose in ISF by coupling MNs with enzymatic glucose biosensors. (Figure 6E) [34]. The hollow silicon MN array was used as an ISF collector. A glucose oxidase based electrochemical chip delicately adhered to the end of MN for measuring glucose in real time. The application of MNs for ISF sampling is an elegant approach to monitor many important health-related parameters in a minimally invasive manner, which presents a great potential for POC application.

### 4.3. Disease Treatment 

#### 4.3.1. Drug Delivery

Abundant improvements have been achieved since MN was first applied to drug delivery in 1998 [135]. With the advancement of new materials, novel fabrication techniques, and creative delivery strategies, MNs technology, which can transdermally deliver drug in a minimally invasive manner, provides a promising method to replace subcutaneous self-injection for disease treatment. Hollow MN as the alternative to subcutaneous needle meets the need of mass transportation or delivery of relative large substances like cells [136]. When it comes to small molecules (e.g., insulin), coated, swellable, or dissolving MNs can be good choices.

**Figure 7:**
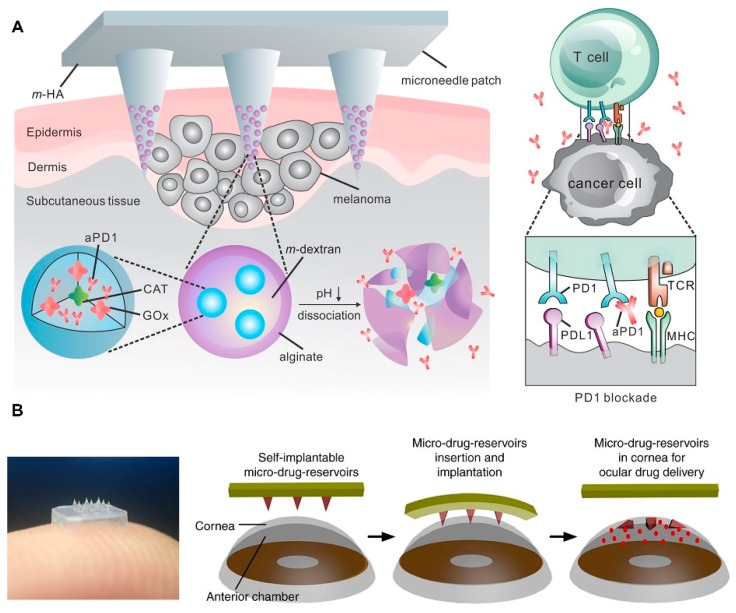
(**A**) Schematic illustration of GOx and catalase (CAT) assisted anti-PD-1 delivery by nanoparticle loaded dissolving MN (reproduced with permission from the authors of [141]). (**B**) Schematic illustration of self-implantable double-layered MN in ocular application and real product photograph. (reproduced with permission from the authors of [144]).

By tailoring the structures, materials, and fabrication methods, an appropriate drug delivery system with good drug load and release kinetic performance can be designed for specific applications. Common control strategies can be divided into three categories: (1) adjusting materials’ dissolution rate for rapid or sustained drug delivery [137,138], (2) multiplying layers for sequenced delivery [139], and (3) integrating well-designed limiter or accelerator into structure for conditional release [140]. In skin cancer immunotherapy, high efficacy, reasonable inhibitor dose, and controllable and sustained drug-releasing mechanism are desirable. Wang et al. demonstrated a nanoparticle-loaded dissolving MN for melanoma therapy (Figure 7A) [141]. Hyaluronic acid (HA) MN pre-loaded pH-sensitive dextran nanoparticles (NPs), which encapsulated GOx and immunomodulator anti-programmed death (PD)-1, was used in melanoma treatment. Released GOx turned blood glucose into gluconic acid, and thus formed an acidic environment, which enhanced self-dissociation of NPs and accelerated subsequent release. With the same dose of drug, these smart MNs induced more intense immune responses than MNs without trigger. Besides, this strategy can integrate with other immunomodulators and further improve antitumor activity. Zhen Gu’s group reported a degradable MN patch consisting of drug-loaded NPs to locally deliver browning reagents for the clinical treatment of obesity [142]. Nanoparticles encapsulating rosiglitazone, glucose oxidase, and catalase were prepared from pH-sensitive acetal-modified dextran and coated with alginate. The NPs were further integrated into an MN-array patch, which was made of cross-linked HA matrix for sustained drug delivery. The degradable NPs released browning agents into the subcutaneous adipose tissue and promoted the transformation of white adipose tissue toward the brown-like adipose tissue, and thus achieved effective obesity treatment. In recent years, MN has begun to be applied to non-transdermal drug delivery in ocular, vascular, oral, and mucosal tissue [143]. As an instance, ocular is sensitive, fragile and has poor drug retention ability. Chen’s group developed an eye patch equipped with an array of detachable MNs for ocular drug delivery (Figure 7B) [144]. They designed a double-layered dissolving MN patch as a self-implantable therapeutic tool to treat corneal neovascularization. The external layer of MN rapidly dissolved and released an anti-inflammatory compound, while the internal part enabled a long-term drug delivery. The biphasic drug release kinetics enhanced therapeutic efficacy, and ~90% neovascular area removal was achieved by 1 µg of the drug, which was far lower than the dosage of eye drops (10 µg) and systemic intraperitoneal injection (1 mg). Meanwhile, the latter two failed to show significant therapeutic effect. MNs with highly biocompatibility, controllable degradation, and good mechanical properties have been developed for transdermal drug delivery by tailoring the unique shapes, materials, and fabrication methods, which have been proven to increase transdermal drug delivery efficiency.

Developing smart MNs in response to external environmental change in a long-acting way is of vast demand. Chen et al. reported a nondegradable, hydrogel-forming MN composed of a semi-interpenetrating network hydrogel for glucose-responsive insulin delivery in a painless, convenient, and safe manner [145]. The needle region was formed by glucose-responsive silk fibroin and phenylboronic acid/acrylamide. The MNs with microporous and interconnected structure after rehydration responded acutely to the change of glucose, and autonomously released insulin on-demand. Yu et al. developed a novel closed-loop glucose-responsive insulin delivering MN that can mimic the function of pancreatic cells [146]. The key glucose-responsive vesicles were self-assembled from hypoxia-sensitive hyaluronic acid (HS-HA) conjugated with 2-nitroimidazole (NI). Insulin and glucose oxidase enzyme were loaded in the glucose-responsive vesicles. When hydrophobic NI was placed in hypoxic conditions, which was conducted by enzymatic oxidation of glucose, it would become hydrophilic and accelerate the reduction of HS-HA, resulting in rapid release of insulin. The blood glucose of type 1 diabetes mouse regulated by this integrated MN remained stable, presenting its feasibility in both sensing and therapy. These smart MNs provide sustained and glucose-responsive insulin delivery, which shows tremendous potential to achieve persistent glycemic management. Various sensors and actuators can be integrated into the MN platform. These MNs can respond to specific external stimuli and administrate drug releasing activity in real time, showing great potential to improve the health of diabetics.

#### 4.3.2. Vaccine Delivery

MN-mediated vaccine delivery has developed rapidly and become one of the most investigated topics among MN applications [147]. A large amount of immune cells, including Langerhans cells (APCs), dendritic epidermal T-cells, and innate lymphoid cells (ILCs), present in the epidermis/dermis [148]. Therefore, MN-based intradermal vaccine administration can induce a dose-sparing effect and stimulate a strong immune response in low dose [149]. Vaccine-loaded MNs are appreciated to control vaccine release kinetics or enhance immune response by tailoring the composites of MNs [150,151]. Li et al. developed a dissolving MN array for ovalbumin (OVA) delivery. Chitosan nanoparticles (NPs) encapsulating OVA, and an adjuvant, oligodeoxynucleotides, were loaded in the tips of the dissolving MN [152]. The chitosan NPs with positive charge can form nanocomplexes with negatively charged antigens and adjuvants, which greatly facilitates the uptake efficiency of DC2.4 cells. Furthermore, the chitosan nanocomplexes accumulated in peripheral lymph nodes of mice, and thus promoted immune response. Using layer-by-layer assembly, Uppu et al. developed polymer multilayer films on microneedle arrays for the assembly of vaccines with tunable release kinetics of different components [153]. Domain III subunit antigen, two adjuvants, and an amphiphilic hexapeptide were enveloped in polymer multilayer coating. The coating films were implanted into the dermis and epidermis after MN removal. Remarkably, releasing profiles of each component could be adjusted from days up to two weeks separately. MN as a promising and robust platform shows great potential for efficient transcutaneous vaccine delivery.

Some MN-mediated vaccines have gone into clinical trials, and begin commercialization processes in recent years. Rouphael et al. have done the first phase 1 human clinical trial of inactivated influenza vaccine using dissolving MN patches [154,155]. Participants were divided into four groups randomly, and received the same dose of inactivated influenza vaccine (1) by an influenza vaccine-containing MN patch (MNP_IIV-HCW_), (2) by an influenza vaccine intramuscular injection (IM_IIV_), (3) received placebo by a placebo-containing MN patch (MNP_placebo_), all administered by a health-care person; or (4) by the vaccine-containing MN patch (MNP_IIV-self_), self-administered by themselves, respectively. MN-mediated vaccine was shown to be well tolerated and caused no serious adverse events. Accordingly, 70% of recipients were likely to use MN-mediated vaccination in the future. The IM_IIV_ and MNP_IIV-HCW_ groups induced similar levels of immune response and had no distinct differences with the results of MNP_IIV-HCW_ and MNP_IIV-self_ groups, indicating the MN patch is a safe, efficient, and user-friendly tool for vaccination. MN-mediated vaccine delivery provides a new approach with the potential to improve immunization effect and reduce immunization costs, which is suitable for elder people [156], compatible with other diseases [157], and feasible for nonhuman primates [158]. Besides clinical trials, many commercial MN products have been developed for vaccination and are available nowadays. These commercial products, for example, Nanopatch^TM^ [159], Fluzone Intradermal^®^ [160], and Intanza^®^ [161], have been published in journals, and contributed to public health against pestilence [162,163].

### 4.4. Microelectronic System Integrated MN Devices for Sensing and Therapy

**Figure 8:**
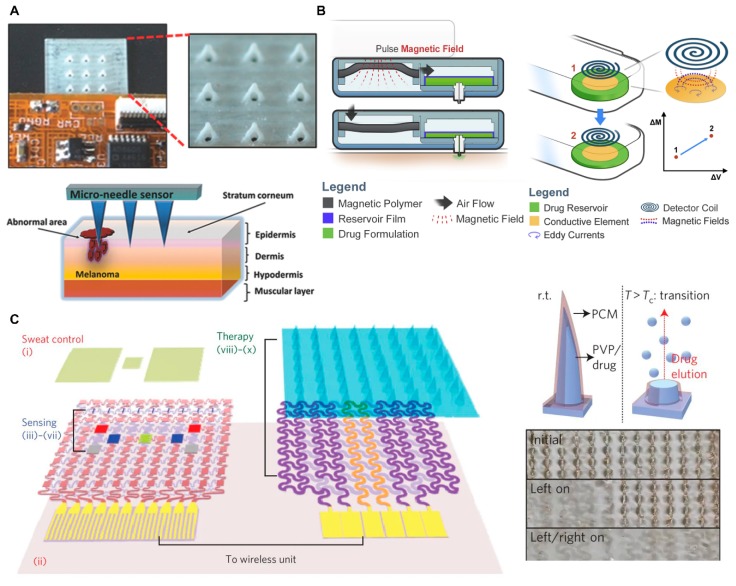
(**A**) Photograph of MN integrated with flexible electronics (top) and schematic illustration of melanoma biomarker detection using MN (down) (reproduced with permission from the authors of [164]). (**B**) Schematic illustration of pneumatic-hydraulic actuator system used for wireless drug delivery (left) and related inductive sensing principal (right) (reproduced with permission from the authors of [165]). (**C**) Schematic illustration of diabetes sensing and therapy patch (left), drug releasing principal of MN (right top), and photograph of heater controlled stepwise MN dissolution (right down) (reproduced with permission from the authors of [166]).

With the advancement of soft electronics, materials, and fabrication techniques, microelectronic system integrated MN devices are emerging, which makes the clinical application of MN move from traditional transdermal sensing and drug delivery to smart diagnosis and therapy. The microelectronic system integrated MN devices can transform biosignals into electrical signals for comprehensive healthcare. Cui et al. described a novel wearable bandage and a MN-based electrochemical sensing device toward rapid screening of skin melanoma (Figure 8A) [164]. The hollow MN sensor was modified with catechol-coated carbon paste for screening tissue tyrosinase levels, a biomarker of skin melanoma. When making contact with tyrosinase, catechol was converted to benzoquinone and produced a measurable amperometric signal. The wearable MN sensor was connected to a flexible electronic board, which could analyze the collected signal and control the wireless data transmission to a mobile device. Microelectronic systems can also transfer other kinds signal to therapeutic action. Jayaneththi et al. developed a wirelessly controlled transdermal drug delivery system that is capable of drug dispensing and dosage sensing (Figure 8B) [165]. The key actuator part of this device was made up of magnetic polymer composite (MPC), which is flexible and magnetically driven. When an external magnetic field switched on, air was forced into drug reservoir by MPC motion, and the drug was delivered through hollow MN to dermal layer without extra power supply. In addition, the total volume dispensed by the drug delivery device was wirelessly measured by inductive sensing. A planar detector coil was placed on top of a copper film attached to the drug reservoir, and centrally aligned with the axis of the copper film. The oscillating magnetic field of the coil can induce eddy currents in the copper film, and produce its own magnetic field. The interaction between the two magnetic fields induces a change in inductance, which can be applied to measure the change in volume. The two microelectronic systems integrated MN devices display attractive analytical performance and offer considerable promise for diagnosis and therapy. New design challenges arise for longitudinal sensing and controlled drug release on dynamic tissue. Dae-Hyeong Kim’s group developed a wearable diabetes patch, which consisted of sweat-control components, sensing components, and therapeutic components (Figure 8C) [166]. Graphene integrated with a gold mesh was applied to form the wearable electrochemical device, which not only monitored pH, temperature, humidity, and glucose level in sweat, but also thermally controlled the release of metformin synchronously by dissolving MNs for diabetes therapy. They further developed a wearable sweat glucose monitoring device integrated with a feedback-controlled MN-based therapy platform [167]. The novel system was composed of humidity, glucose, pH, temperature sensors, and feedback-controlled MNs. The sensors of the device measured sweat glucose, which is correlated with blood glucose level. Upon hyperglycemia, thermal actuation controlled the transdermal MN-based drug delivery without pain and stress. The novel system provides a high-fidelity sweat glucose measurement for noninvasive sweat-based management of diabetes mellitus in response to the patient’s glucose level. The combination of biomarkers and physiological cues monitoring with transdermal MN-based drug delivery achieved a closed-loop, painless, point-of-care treatment for diabetes. For practical application of these systems, the long-term stability, fabrication cost, and uniformity of the devices are of great importance to enable the integrated system to be practically applicable to clinics.

## 5. Summary and Outlook

As cost-effective, reliable, minimally invasive devices, MNs conquered much more space in the therapy and diagnostics of diseases by facilitating the dynamic dosing of therapeutics, and monitoring of biomarkers of clinical interests both in blood and ISF. The MN devices can be easily obtained by changing the fabrication technology, materials, and production parameters, which can be classified into solid, hollow, coated, dissolving, and swellable MNs. Different types of MNs possess various physical and chemical characteristics, such as conductivity, morphology, wettability, swellability, and biodegradability. MNs have shown promising preclinical outcomes and are expected to contribute to personalization of treatment and management of chronic diseases. MN-based POC devices for vaccination against influenza are already commercial [168]. Hollow and solid MNs systems (e.g., Soluvia™ microinjection, MicronJet™) are clinically approved for transdermal drug delivery, while most of MNs for therapeutics and diagnosis are still in the development phase. 

Future clinical analysis devices are expected to minimize the labor cost and eliminate the restriction of operational site, thereby enabling common citizens at any cities and any levels to obtain medical care with negligible costs [169]. Despite many progresses on MNs fabrication, current MN technology suffers from limited functional surface area or challenges in the scale-up of fabrication. The micro size of MN endows it with painlessness, while resulting in limited functional surface area for sensing and therapy. Porous, swellable, hollow MNs have a relatively larger functional surface area compared with coated and solid MNs to some extent. It is wise to choose an appropriate type of MNs and design the structure according to the specific application. Appropriate fabrication methods should be applied to gain sensitive drug-loaded MNs, because harsh processing conditions (e.g., heat and light irradiation) will affect the activity of sensitive drugs. Recently, Kim et al. designed a self-administrative powder-carrying MN to directly implant insulin powder inside skin [170]. A dissolving MN array with empty cavities in the center was prepared by a mold-casting method. Insulin powder was loaded into the cavities and sealed by carboxymethyl cellulose film. Because no drug reconstitution step was involved in the fabrication, the MNs’ design overcame the loading capacity and activity loss during MN fabrication. The powder-carrying MNs showed enhanced long-term stability and prolonged release kinetics without apparent safety issues, which showed great potential for long-term diabetes treatment. Convenient, cost-effective, mild, and efficient manufacturing processes are highly desired for the fabrication of MN devices with good biocompatibility, no risk of infection, and painlessness. In practice, good skin penetration ability, painlessness, high sensitivity, good drug release, and minimal invasiveness are necessary requirements for MNs. Multifunctional MN theranostic devices can be achieved by integrating transdermal biosensing and drug delivery. They can accurately monitor analytes of clinical significance in the skin or administer treatments according to drug/metabolite concentrations in response to precision delivery. With the advent of advances in micro-electromechanical systems, 3D printing, remote signal monitoring technology, and microfluidic chip, MN-based POC systems hold enormous potential for growth driven by both clinics and market requests.

## Figures and Tables

**Table 1:** Materials for fabrication of microneedles (MNs).

Materials	Advantages	Disadvantages	Application	Reference
Silicon	Hard, Mature fabrication techniques, Biocompatible	Brittle, Easy to produce sharp waste	Solid, Coated, Hollow MNs	[33,34,35]
Glass	Transparent, Chemical inert, and Low-cost	Brittle, Cumbersome fabrication	Hollow MNs	[36,37,38]
Ceramics	Natural porous	Significant brittle fracture, Long fabrication time	Hollow, Dissolving MNs	[39,40,41]
Metal	High conductivity, Biocompatibility, and Some nanostructured metals have catalytic activity	Allergenic risk, High cost for noble metals	Solid, Coated, Hollow MNs	[42,43,44,45,46]
Polymers	Easy fabrication, Some are biodegradable or swellable	Low mechanical strength	Solid, Hollow, Coated, Dissolving, Swellable MNs	[17,47,48,49,50]
Carbohydrate	Biodegradable, Biocompatible	Low mechanical strength, High processing temperatures and hygroscopicity	Dissolving MNs	[26,51,52]
